# Posterior Dislocation of the Long Head of Biceps Tendon after Traumatic Anterior Shoulder Dislocation

**DOI:** 10.5334/jbsr.3515

**Published:** 2024-03-04

**Authors:** Marlena Bereźniak, Piotr Palczewski, Paweł Łęgosz

**Affiliations:** 1 1st Department of Clinical Radiology, Medical University of Warsaw, Warsaw, Poland; 2 1st Department of Clinical Radiology, Medical University of Warsaw, Warsaw, Poland; 3 Department of Orthopaedics and Traumatology of the Locomotor System, Medical University of Warsaw, Warsaw, Poland

**Keywords:** shoulder dislocation, biceps tendon injuries, diagnostic imaging

## Abstract

*Teaching point:* An irreducible anterior glenohumeral joint dislocation associated with a displaced fracture of greater tuberosity, a rotator cuff tear, or a coracoid process fracture should raise the suspicion of posterior long head of biceps tendon (LHBT) dislocation.

## Case

**Figure 1:**
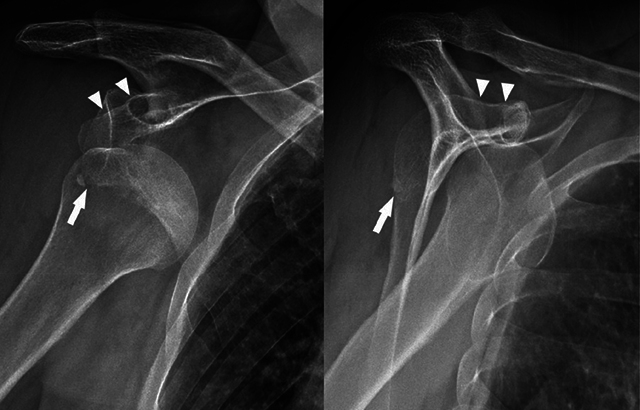
Anteroposterior and trans-scapular Y radiographs of the right shoulder demonstrating anterior subcoracoid dislocation of the humeral head (arrowheads) and displaced inferior glenoid rim fracture (arrows).

**Figure 2:**
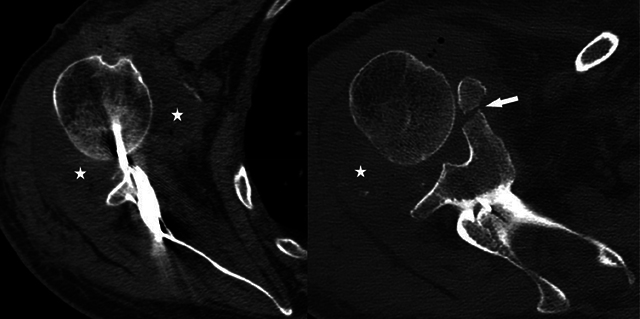
Postoperative axial CT scans of the right shoulder showing displaced coracoid process fracture (arrow), glenohumeral joint effusion (asterisks), and anterior humeral head subluxation.

**Figure 3:**
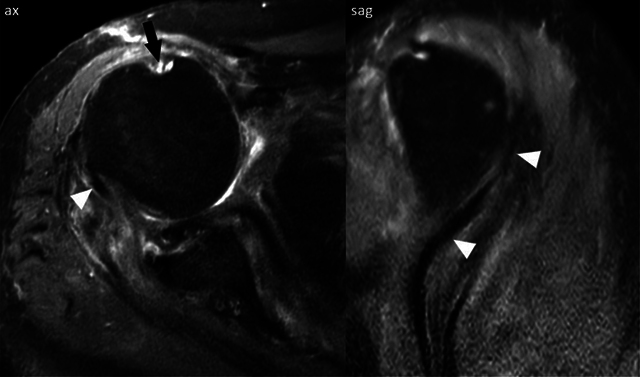
Axial and sagittal fat-saturated proton density-weighted MRI images showing a complete rotator cuff tear, an empty bicipital groove (arrow), and dislocation of the intact long head of biceps tendon (LHBT) posteriorly to the humeral head (arrowheads).

A 76-year-old male presented to the emergency department (ED) with pain and reduced range of motion in the right shoulder after a fall from stairs. On physical examination, there was a marked soft tissue swelling and the right arm was slightly abducted and externally rotated. The neurovascular examination was normal. The initial radiograph (anteroposterior and trans-scapular Y views) showed anterior subcoracoid dislocation of the humeral head (Figure 1, arrowheads) and displaced inferior glenoid rim fracture (Figure 1, arrows). The patient was treated unsuccessfully with closed manipulative reduction, so open reduction with Kirschner wire fixation was pursued. A postoperative computed tomography (CT) scan revealed a displaced fracture of the coracoid process (Figure 2, arrow), glenohumeral joint effusion (Figure 2, asterisks), and anterior humeral head subluxation. A magnetic resonance imaging (MRI) performed to identify causes of irreducible shoulder dislocation showed a complete rotator cuff tear, an empty bicipital groove (Figure 3, arrow), and dislocation of the intact long head of biceps tendon (LHBT) posteriorly to the humeral head (Figure 3, arrowheads).

## Comment

Anterior shoulder dislocation is the most frequent large joint dislocation encountered in the ED. Associated injuries include greater tuberosity fracture, Hill-Sachs and/or bony Bankart lesions, glenoid labrum injuries, and rotator cuff tears. Lateral or posterior dislocation of LHBT is a rare complication, usually following a high-energy trauma. It occurs secondary to a disruption of the biceps pulley and lateral stabilizing structures of the glenohumeral joint with fracture of the greater tuberosity and/or a complete tear of the supraspinatus and infraspinatus tendons [1]. The interposition of LHBT posterior to the humeral head mechanically prevents a closed reduction and requires an open reduction with tenotomy and tenodesis of LHBT.

Standard radiographic examination or CT scans should raise the suspicion of posterior LHBT dislocation when the humeral head is displaced medially to the coracoid process or a large fractured fragment of the greater tuberosity is displaced more than 1 cm [1]. The association of anterior shoulder dislocation and coracoid fracture is unusual. In this case, the fracture of the coracoid process was most probably caused by the direct impact of a medially displaced humeral head. Although a dislocation of the humeral head medially to the coracoid process is rarely seen on radiographs, an anterior shoulder dislocation associated with coracoid process fracture should suggest posterior LHBT dislocation in case of unsuccessful closed reduction [1].
